# Sustained efficacy and safety of repeated incobotulinumtoxinA (Xeomin^®^) injections in blepharospasm

**DOI:** 10.1007/s00702-013-0998-9

**Published:** 2013-02-23

**Authors:** Daniel D. Truong, Stephen M. Gollomp, Joseph Jankovic, Peter A. LeWitt, Michael Marx, Angelika Hanschmann, Hubert H. Fernandez, Xeomin US Blepharospasm Study Group

**Affiliations:** 1 The Parkinson’s and Movement Disorder Institute, 9940 Talbert Avenue, Fountain Valley, California, 92708 USA; 2 Division of Neurology, Lankenau Hospital, Wynnewood, PA USA; 3 The Parkinson’s Disease and Movement Disorders Clinic, Department of Neurology, Baylor College of Medicine, Houston, TX USA; 4 Department of Neurology, Henry Ford Hospital, Detroit, MI USA; 5 Merz Pharmaceuticals GmbH, Frankfurt am Main, Germany; 6 Movement Disorders, Center for Neurological Restoration, Cleveland Clinic, Cleveland, OH USA

**Keywords:** Blepharospasm, Botulinum toxin, IncobotulinumtoxinA, Dystonia, Xeomin

## Abstract

IncobotulinumtoxinA (Xeomin^®^, NT 201) is a purified botulinum toxin type A free from accessory (complexing) proteins. Previous studies evaluated single sets of incobotulinumtoxinA injections for the treatment of blepharospasm. Individualized injection intervals and other potential determinants of efficacy and safety need to be evaluated in a prospective, longitudinal study. Subjects with blepharospasm who completed a ≤20 weeks double-blind, placebo-controlled main period entered a ≤69 weeks open-label extension period (OLEX) and received ≤5 additional incobotulinumtoxinA treatments at flexible doses (≤50 U per eye) and flexible injection intervals (minimum of 6 weeks). Outcome measures included Jankovic Rating Scale (JRS) (sumscore, severity subscore and frequency subscore), Blepharospasm Disability Index, and adverse events. All 102 subjects who completed the main period entered the OLEX; 82 subjects completed the study, 56 received the maximum five injections. From each injection visit to a control visit 6 weeks later, investigator-rated JRS sumscores and subscores, and patient-rated Blepharospasm Disability Index were significantly improved (*p* ≤ 0.001 for all). All scores were still significantly improved at trial termination compared with the first injection visit (*p* < 0.05 for all). The most frequently reported adverse events were eyelid ptosis (31.4 %) and dry eye symptoms (17.6 %). The injection interval had no impact on the incidence of adverse events (post hoc analysis). No subject developed neutralizing antibodies during the study. Repeated incobotulinumtoxinA injections, administered at flexible doses and injection intervals from 6 to 20 weeks according to subjects’ needs, provide sustained efficacy in the treatment of blepharospasm with no new or unexpected safety risks.

## Introduction

Blepharospasm is a focal dystonia characterized by excessive involuntary contractions of the muscles surrounding the eyes (Hallett et al. 2008). Patients experience a reduced quality of life and, in severe cases, can even suffer from functional blindness (Daly 1997).

Botulinum toxin type A has been successfully used for the treatment of blepharospasm for more than 20 years (for review see Truong and Jost 2006), having demonstrated efficacy in several controlled clinical trials (Jankovic and Orman 1987; Jankovic et al. 2011; Roggenkämper et al. 2006; Truong et al. 2008). IncobotulinumtoxinA (Xeomin^®^; also known by its internal drug code NT 201; Merz Pharmaceuticals GmbH, Frankfurt am Main, Germany) is a highly purified, lyophilized botulinum neurotoxin type A formulation. When isolated from *Clostridium botulinum* cultures, botulinum toxin is a protein complex consisting of the 150 kDa core neurotoxin and accessory (complexing) proteins (Inoue et al. 1996). As a result of a unique purification process, incobotulinumtoxinA contains only the 150 kDa neurotoxin, and unlike other botulinum toxin formulations is free from accessory (complexing) proteins (Frevert 2009; Frevert 2010; Frevert and Dressler 2010). IncobotulinumtoxinA has demonstrated efficacy and safety comparable to onabotulinumtoxinA (Allergan Inc., Irvine, CA, USA) in the treatment of blepharospasm (Roggenkämper et al. 2006) and cervical dystonia (Benecke et al. 2005) when the same unit doses were used.

In a double-blind, randomized, placebo-controlled study, treatment with incobotulinumtoxinA demonstrated superiority versus placebo for patients with blepharospasm (Jankovic et al. 2011). As blepharospasm is a chronic condition, the investigation of long-term treatment options is essential. Here, we present data from the open-label extension period (OLEX) of the placebo-controlled study to evaluate the safety and efficacy of repeated injections of incobotulinumtoxinA in the treatment of blepharospasm. The study design incorporated flexible dosing and flexible injection intervals to allow tailoring of treatment to the needs of the individual patients.

## Methods

The results of the preceding double-blind, randomized, parallel-group, placebo-controlled main period (MP; clinicaltrials.gov identifier NCT00406367) of the trial have been reported previously with the corresponding inclusion and exclusion criteria (Jankovic et al. 2011). The OLEX had an unblinded, non-controlled design and was conducted at 34 centers in the US and Canada. The responsible Institutional Review Boards approved the study protocol and informed consent form; patients provided written informed consent. The ethical principles outlined in the Declaration of Helsinki and Good Clinical Practice were followed. The study was monitored by an independent Data Safety Monitoring Board.

### Subjects

Subjects enrolled in this study had completed the MP, and had expressed the need for a new injection, confirmed by the investigator [defined as a Jankovic Rating Scale (JRS) severity subscore ≥2]. Prior to the MP, all subjects had received at least two treatments with onabotulinumtoxinA. The doses used in these onabotulinumtoxinA injections were the basis for the dose of incobotulinumtoxinA administered during the MP (Jankovic et al. 2011), using a clinical conversion ratio 1:1 between onabotulinumtoxinA and incobotulinumtoxinA (Roggenkämper et al. 2006). Re-injection during the OLEX was possible from as early as 6 weeks up to the time whenever the patient expressed the need for a new injection. There were no specific exclusion criteria for the OLEX.

### Treatment

During the OLEX, subjects could receive a maximum of five incobotulinumtoxinA injections over ≤49 weeks, followed by a safety observation period of ≤20 weeks (total duration ≤69 weeks). In standard clinical practice, the treatment interval is typically restricted to around 12 weeks based on the presumption that this delay will lessen the chance of antibody formation against botulinum toxin. However, this study employed flexibility in dosing and intervals, enabling investigators to re-inject based on subjects’ needs. Subjects had to contact the investigator to request a re-injection; re-injection criteria included a ≥6-week injection interval and a JRS severity subscore ≥2. Dose, dilution, number of injections, and injection sites were flexible and tailored to each individual subject by the investigator, based on the severity and frequency of spasms, individual response, and history of adverse events (AEs) of each subject. The total maximum dose per injection session was 100 U (50 U per eye).

Each injection visit was followed by an office visit 6 weeks later when symptoms were assessed. The trial termination visit (TTV) took place 20 weeks (±3 days) after the last injection or when the subject asked for a new injection after the end of the 49 weeks treatment period, whichever came first.

### Efficacy assessments

#### Jankovic Rating Scale

Severity and frequency of blepharospasm symptoms were measured using the JRS, which is scored on a scale 0–8 points (sumscore) and includes two subscores: severity and frequency, both ranging from 0 to 4 (Jankovic and Orman 1987; Jankovic et al. 2009). JRS scores were assessed at all visits by trained and certified investigators. Changes in mean JRS scores from each injection visit to the respective control visit 6 weeks later, and from the first and the last injection visit to the TTV, were analyzed.

#### Blepharospasm Disability Index

Functional impairment was assessed using the Blepharospasm Disability Index (BSDI), a self-rating scale that includes six daily activity items (“driving a vehicle”, “reading”, “watching TV”, “shopping”, “walking”, “doing everyday activities”) (Roggenkämper et al. 2006; Jankovic et al. 2009). These items were rated on a five-point scale ranging from 0 (no impairment) to 4 (no longer possible due to my illness). Patients were permitted to rate items as “not applicable” (except “doing everyday activities”). The BSDI mean score is the sumscore of all applicable items, divided by the number of applicable items. Changes in BSDI mean scores were analyzed from each injection visit to the respective control visit 6 weeks later, and from the first and the last injection visit to the TTV.

#### Patient Evaluation of Global Response (PEGR)

Subjects described their global response using a nine-point scale ranging from −4 (very marked worsening) to +4 (complete abolition of all signs and symptoms) (adapted from Wissel et al. 2000) at all injection visits (except the first), and at the TTV.

### Safety assessments

Throughout the study, subjects were requested to report all AEs to the investigator. Additionally, they were asked specifically at all visits about the occurrence of AEs that could indicate distant effects from toxin spread, such as stomach and bowel disturbances, drooping of eyelids, vision problems, dry mouth, swallowing difficulties, speech problems, shortness of breath, respiratory infection, local weakness, facial weakness, and general body weakness. Physical and neurological examinations were conducted at the beginning of the OLEX, the third injection visit, and the TTV. Blood samples for laboratory tests and determination of antibodies against botulinum toxin were collected at all injection visits and the TTV. Samples were initially screened for botulinum neurotoxin antibodies using a fluorescence immunoassay (FIA); as the FIA cannot discriminate between neutralizing and non-neutralizing antibodies, positive FIA samples were subsequently tested using a mouse hemidiaphragm assay (HDA) (Göschel et al. 1997; Sesardic et al. 2004).

Investigators rated the tolerability of incobotulinumtoxinA at all injection visits (except the first), and at the TTV, using a four-point scale ranging from 1 (very good) to 4 (poor), based on patient reports.

### Statistical analysis

All efficacy variables were analyzed in the intent-to-treat (ITT) population (all subjects who were randomized in the MP and included in the OLEX). Changes in mean JRS scores and BSDI mean score were analyzed with one-sample *t* tests with no replacement of missing data. Safety analyses were carried out in the evaluable-for-safety (EFS) population (all subjects who received ≥1 incobotulinumtoxinA injection during the OLEX). AEs were encoded using the Medical Dictionary for Regulatory Activities (MedDRA), Version 9.1.

In a post hoc analysis, a Chi-square test was used to compare the overall occurrence of AEs between groups of patients with different median injection intervals (6 to ≤10 weeks, >10 to ≤12 weeks, >12 to ≤14 weeks, or >14 weeks). All statistical analyses were performed using SAS version 8.2 or later (SAS Institute, Cary, NC, USA).

## Results

### Subjects

One-hundred and two subjects with blepharospasm completed the MP and all continued into the OLEX. The first subject entered the OLEX on December 12, 2006 and the last subject completed the study on July 14, 2009. Eighty-two subjects (80.4 %) completed the OLEX; 20 subjects (19.6 %) discontinued prematurely due to withdrawal of consent (*n* = 6), insufficient efficacy (*n* = 4), protocol violations (*n* = 4), loss to follow-up (*n* = 2), or occurrence of withdrawal criteria (*n* = 4), which included eyelid surgery (*n* = 2), treatment with a different botulinum toxin type A before the TTV (*n* = 1), and the need for general anesthesia (*n* = 1). For the latter subject, breast cancer was documented as an additional reason for withdrawal, representing the only discontinuation due to an AE; no subjects discontinued due to an adverse drug reaction (ADR). Of the subjects who discontinued due to insufficient efficacy, two returned to onabotulinumtoxinA injections (after receiving two and three incobotulinumtoxinA treatments in the OLEX, respectively), one felt that incobotulinumtoxinA was effective but that the minimum treatment interval of 6 weeks was too long, and no further details were documented for the fourth subject. Baseline characteristics are shown in Table 1.

**Table 1:** Characteristics of subject population at the OLEX baseline (ITT population)

	Total (*n* = 102)
Male gender, *n* (%)	36 (35.3)
Race, *n* (%)	
Asian	6 (5.9)
Black or African American	4 (3.9)
Hispanic or Latino	8 (7.8)
White	84 (82.4)
Mean age, years (SD)	62.2 (10.3)
Mean BMI, kg/m^2^ (SD)^a^	28.4 (5.3)
Mean duration since first diagnosis of blepharospasm, months (SD)^b^	65.9 (61.1)
Mean estimated duration of blepharospasm, months (SD)^b^	106.6 (90.0)
Mean JRS scores (SD)	
JRS sumscore	5.9 (1.4)
JRS severity subscore	3.1 (0.8)
JRS frequency subscore	2.8 (0.8)
Mean BSDI (SD)	1.50 (0.83)
Most frequent (≥40 subjects) concomitant diseases, *n* (%)	
Dry eye	56 (54.9)
Eyelid ptosis	40 (39.2)

*BMI* body mass index, *BSDI* Blepharospasm Disability Index, *ITT* intent-to-treat, *JRS* Jankovic Rating Scale, *MP* main period, *OLEX* open-label extension period, *SD* standard deviation

^a^Height to calculate the BMI was assessed at screening for the MP

^b^At screening for the MP

All subjects who entered the OLEX received ≥1 incobotulinumtoxinA injection. The mean [standard deviation (SD)] injection interval during the OLEX was 12.6 (4.5) weeks (median 12 weeks); 94.9 % (392/413) of re-injections were administered at intervals of ≥6 to ≤20 weeks. For the 93 subjects who received ≥2 injections, the median injection interval was 6 to ≤10 weeks for 22 subjects (23.7 %), >10 to ≤12 weeks for 30 subjects (32.3 %), >12 to ≤14 weeks for 23 subjects (24.7 %), and >14 to 20 weeks for 18 (19.4 % of subjects) (Table 2). Fifty-six subjects (54.9 %) received all five injections and 25 subjects (24.5 %) received four injections. The mean (SD) dose of incobotulinumtoxinA administered for both eyes ranged from 64.7 (22.4) U at the first injection visit to 72.7 (22.0) U at the fifth visit; the overall range of doses was 15.0–100.0 U. The mean duration of the OLEX was 52.6 weeks (range 6.3–75.0 weeks).

**Table 2:** Median injection intervals and incidence of AEs by injection group in subjects with ≥2 injection visits in the OLEX (post hoc analysis)

Median injection interval	Number of subjects in interval group^a^ *n* (%)	Number of subjects with ≥1 AE^b^ *n* (%)
6 to ≤10 weeks	22 (23.7)	18/22 (81.8)^c^
>10 to ≤12 weeks	30 (32.3)	24/30 (80.0)^c^
>12 to ≤14 weeks	23 (24.7)	22/23 (95.7)^c^
>14 to 20 weeks	18 (19.4)	12/18 (66.7)^c^
All intervals	93 (100)	76/93 (81.7)^c^

Subjects were actively asked at each visit if they experienced drooping of the eyelid, problems with vision, dry eyes, dry mouth, swallowing difficulties, speech problems, shortness of breath, respiratory infection, local weakness, facial weakness, general body weakness, or stomach or bowel disturbances

*AE* treatment-emergent adverse event, *OLEX* open-label extension period

^a^Ninety-three subjects received ≥2 injections and ≤5 injections in the OLEX and were included in this analysis

^b^Seventy-six of these 93 subjects experienced ≥1 AE during the OLEX

^c^A Chi square test did not show significant differences in the overall occurrence of AEs between the different injection interval groups (*p* = 0.1229)

### JRS sumscore and subscores

**Fig. 1:**
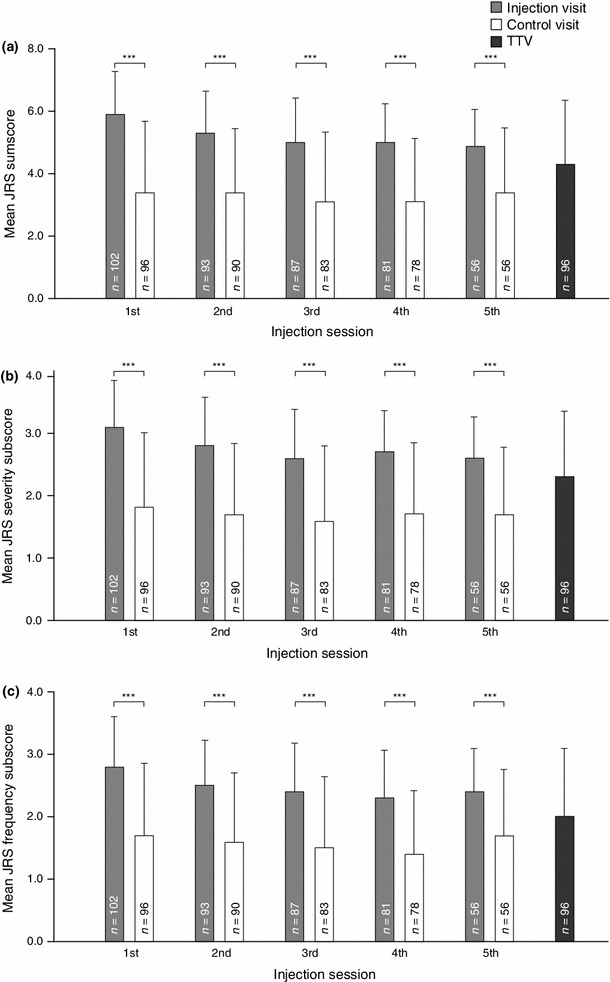
Mean JRS sumscore (**a**), severity subscore (**b**), and frequency subscore (**c**) at injection visits, control visits 6 weeks following injection visits, and the TTV (ITT population). *ITT* intent-to-treat, *JRS* Jankovic Rating Scale, *TTV* trial termination visit ***p < 0.001, one-sample *t* test, for the change from the injection visit to the respective control visit 6 weeks later (calculated only for subjects who attended both the injection and the respective control visit).* Error bars* represent the standard deviation. The TTV took place between 6 and 20 weeks after the last injection visit

Mean JRS sumscores significantly improved from each injection visit to the respective control visit 6 weeks later (*p* < 0.001 for all visits; Fig. 1a), with mean (SD) differences between each control and respective injection visit ranging from −1.6 (1.8) to −2.4 (2.2). Mean (SD) JRS sumscores at the injection visits decreased from 5.9 (1.4) at the first injection visit to 4.9 (1.2) at the fifth injection visit. Mean (SD) JRS sumscores at control visits ranged from 3.1 (2.0) to 3.4 (2.3). At the TTV, the mean JRS sumscore was significantly reduced from the first and the last injection visit (*p* < 0.001). The improvement in the JRS sumscore from the first injection visit to the control visit 6 weeks later was slightly higher for subjects who had received incobotulinumtoxinA during the MP compared to subjects who had received placebo in the MP (−2.5 [2.0] vs. −2.2 [2.7]). However, differences between the two treatment groups of the MP were not statistically significant at the end of the OLEX.

The mean JRS severity (Fig. 1b) and frequency (Fig. 1c) subscores followed a similar pattern to the mean JRS sumscore, indicating significant improvements between each injection visit and the respective control visit 6 weeks later (*p* < 0.001 for all injection visits). Both subscores were significantly reduced from the first and the fifth injection visit to the TTV (*p* ≤ 0.002 for all).

### Blepharospasm Disability Index

**Fig. 2:**
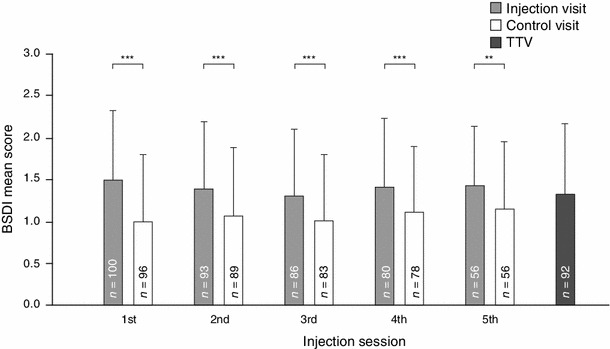
BSDI mean score at injection visits, control visits 6 weeks following injection visits, and the TTV (ITT population). *BSDI* Blepharospasm Disability Index, *ITT* intent-to-treat, *TTV* trial termination visit **p = 0.001, ***p < 0.001, one-sample *t* test for the change from the injection visit to the respective control visit 6 weeks later.* Error bars *represent the standard deviation. The TTV took place between 6 and 20 weeks after the last injection visit

Six weeks after each injection visit, the BSDI mean score was significantly improved (*p* ≤ 0.001 for all injection visits; Fig. 2). Mean (SD) difference in the BSDI mean score between each control and the respective injection visit ranged from −0.27 (0.59) to −0.50 (0.67). Improvements from each injection visit to the respective control visit were significant for each single item score of the BSDI (*p* ≤ 0.038 for all). The BSDI mean score was significantly improved from the first injection visit to the TTV (*p* = 0.043).

### Patient Evaluation of Global Response

At least a moderate improvement in PEGR (≥2 points) was documented by the majority of subjects for injection cycles 1–4 and at the TTV (71/93 [76.3 %], 67/87 [77.0 %], 63/81 [77.8 %], 43/56 [76.8 %], and 76/96 [79.2 %], respectively). At the end of each injection cycle, between 5.4 and 11.8 % of subjects reported complete abolition of all signs and symptoms, while 3.3–7.4 % of subjects reported that their symptoms had worsened.

### Adverse events

The most frequently reported AEs were eyelid ptosis and dry eye symptoms, which occurred in 32 (31.4 %) and 18 (17.6 %) subjects, respectively (Table 3). Frequencies of ADRs per injection cycle ranged from 7.1 (4/56) to 11.8 % (12/102) for eyelid ptosis and from 3.6 (2/56) to 6.9 % (7/102) for dry eye symptoms. In total, 44 subjects (43.1 %) reported ≥1 ADR over all five injection visits during the OLEX. Most ADRs were of mild (39 subjects [38.2 %]) or moderate (14 subjects [13.7 %]) intensity. Severe ADRs were reported in four subjects (3.9 %) and included eyelid ptosis in three subjects (2.9 %) and dry eye symptoms in one subject (1.0 %). The majority of ADRs was transient and resolved by the end of the OLEX; 12 subjects (11.8 %) experienced ADRs that were not yet fully recovered at study termination. The most common unrecovered ADRs were eyelid ptosis (four subjects, 3.9 %) and dry eye symptoms (five subjects, 4.9 %); they were classed as ongoing but stable by the respective investigators at trial termination. Of note, there appeared to be no trend towards an increase or decrease in the incidence of ADRs with repeated injections (data not shown). Ten patients experienced serious AEs during the OLEX, none of which were considered to be drug-related by the investigators.

**Table 3:** AEs affecting ≥5 % of subjects over the duration of the OLEX with ≤5 injection visits (EFS population)

AE, *n* (%)	Total (*n* = 102)
Subjects with ≥1 AE	81 (79.4)
Eyelid ptosis^a^	32 (31.4)
Dry eye^a^	18 (17.6)
Nasopharyngitis^a^	9 (8.8)
Visual disturbance^a^	8 (7.8)
Upper respiratory tract infection^a^	8 (7.8)
Blurred vision^a^	7 (6.9)
Muscular weakness^a^	7 (6.9)
Asthenia	6 (5.9)
Dyspnea^a^	6 (5.9)

*AE* treatment-emergent adverse event, *EFS* evaluable-for-safety, *OLEX* open-label extension period

^a^Denotes an AE of special interest. Subjects were actively asked at each visit if they experienced drooping of the eyelid, problems with vision, dry eyes, dry mouth, swallowing difficulties, speech problems, shortness of breath, respiratory infection, local weakness, facial weakness, general body weakness, or stomach or bowel disturbances

Physical and neurological examinations and laboratory analyses during the OLEX did not reveal any clinically relevant changes.

### Post-hoc analysis

Table 2 shows the incidence of AEs for the 93 subjects who received ≥2 injections stratified into 4 different injection interval groups. Overall, 76 subjects in this population (81.7 %) experienced at least 1 AE over the duration of the study. A Chi square test did not show significant differences in the overall occurrence of AEs between the different injection interval groups (*p* = 0.1229).

### Neutralizing antibodies

No subject developed neutralizing antibodies, defined by a positive HDA, to incobotulinumtoxinA during the OLEX.

### Global Assessment of Tolerability by investigator

The investigator classified the tolerability of study medication as “good” or “very good” for 91/93 (97.9 %), 85/87 (97.7 %), 79/81 (97.5 %), 54/56 (96.4 %), and 92/95 (96.8 %) of subjects after injection cycles 1–4 and at the TTV, respectively.

## Discussion

This open-label extension of a randomized, placebo-controlled, double-blind study with a duration of up to 89 weeks (MP plus OLEX) demonstrates that repeated injections of incobotulinumtoxinA, administered at flexible intervals with a minimum of 6 weeks and with flexible doses of up to 50 U per eye, are efficacious and well tolerated in the long-term treatment of blepharospasm. This is the first randomized trial in which flexible injection intervals were used in registration trials in the evaluation of the efficacy and safety of botulinum neurotoxin type A.

Six weeks after each injection visit, there were significant improvements in the investigator-rated severity and frequency of blepharospasm symptoms (JRS scores) and significant reductions in functional impairments assessed using the patient-rated BSDI, results similar to those of the MP (Jankovic et al. 2011; Roggenkämper et al. 2006).

Over the course of the study, the mean JRS baseline scores at the injection visits gradually decreased and significant improvements were seen from the first and the fifth injection visit to the TTV. This suggests cumulative and sustained improvements in subjects treated with incobotulinumtoxinA in this long-term study. Moreover, the flexible dosing intervals might have allowed patients to receive a new injection before the treatment effect of the previous incobotulinumtoxinA injections had completely waned. The mean treatment effect, as assessed via JRS scores 6 weeks after each injection visit, remained constant throughout the OLEX.

The trial design permitted flexible treatment intervals with a minimum of 6 weeks. Current US Prescribing Information for onabotulinumtoxinA and incobotulinumtoxinA (both approved for the treatment of blepharospasm by the US Food and Drug Administration) recommend a minimum treatment interval of 12 weeks (Allergan 2011; Merz Pharmaceuticals GmbH 2011), due to concerns that shorter intervals could promote the formation of neutralizing antibodies. However, previous studies with incobotulinumtoxinA in other indications suggest that incobotulinumtoxinA with its low foreign protein content and high specific biological activity is associated with low immunogenicity (Comella et al. 2011; Kañovský et al. 2009, 2011). These data suggested that flexible injection intervals could be applied to allow for treatment individualization based on the individual patient’s clinical needs. In the current study, post hoc analysis did not show that the incidence of AEs differed significantly between subjects with different median injection intervals, suggesting that shorter injection intervals are not more likely to be associated with safety concerns. However, it should be noted that patient groups in the post hoc analysis were relatively small. A recent multi-national survey among 136 patients with CD who received treatment with onabotulinumtoxinA or abobotulinumtoxinA indicated that 78 % of patients preferred injection intervals ≤12 weeks, with 46 % of patients stating they would prefer injection intervals ≤10 weeks (Sethi et al. 2012).

Subjects were specifically questioned about AEs that would indicate toxin spread, including eyelid ptosis and dry eye symptoms, which might have prompted a greater level of reporting. This could have contributed to the seemingly higher incidence of ptosis and dry eye symptoms in this study compared to other reports of botulinum toxin treatment effects in blepharospasm, though these studies also only covered one treatment cycle typically without active questioning (Jankovic and Orman 1987; Roggenkämper et al. 2006; Truong et al. 2008; Wabbels et al. 2011; Allergan 2011). Specific questioning allowed us to monitor for changes in the incidence of ptosis and dry eye symptoms during the succeeding treatment cycles. No trend was noted towards an increase in the incidence of AEs with repeated incobotulinumtoxinA treatments, indicating that repeated injections at flexible intervals between 6 and 20 weeks are a viable long-term treatment option for subjects with blepharospasm. The most frequently observed ADRs, eyelid ptosis and dry eye symptoms, well-known side effects of all botulinum toxin preparations in this indication, were transient, similar to published experience (Allergan 2011; Merz Pharmaceuticals GmbH 2011; Kenney and Jankovic 2008). After each injection cycle, >96 % of investigators reported that incobotulinumtoxinA was “well” tolerated or “very well” tolerated.

Of note in this study, no subjects developed neutralizing antibodies, determined by the in vitro HDA (Göschel et al. 1997), during the MP (Jankovic et al. 2011) or the OLEX. This is consistent with other studies of incobotulinumtoxinA (Kañovský et al. 2009, 2011) and supports accumulating evidence that incobotulinumtoxinA is associated with low immunogenicity.

In conclusion, this OLEX of a double-blind, placebo-controlled study demonstrated that repeated injections of incobotulinumtoxinA at flexible intervals provided sustained efficacy in the long-term treatment of blepharospasm. There were no new or unexpected AEs during this trial and no subjects discontinued due to an ADR. A post hoc analysis by injection interval (6–20 weeks) did not show that the incidence of AEs varied significantly for patients who received repeated injections with different median intervals. Further long-term observation of a large number of subjects over many years will be required to fully evaluate the immunogenicity of botulinum toxin preparations for patients with blepharospasm.

## Study investigators

Richard Barbano (Rochester, NY, USA); Allison Brashear (Winston-Salem, NC, USA); Matthew Brodsky (Portland, OR, USA); Mahan Chehrenama (Alexandria, VI, USA); Cynthia Comella (Chicago, IL, USA); Paul Cullis (Detroit, MI, USA); Fabio Danisi (Kingston, NY, USA); Richard Dubinsky (Kansas City, KA, USA); Aaron Ellenbogen (Bingham Farms, MI, USA); Marian Evatt (Atlanta, GA, USA); Virgilio Gerald Evidente (Scottsdale, AZ, USA); Hubert Fernandez (Gainesville, FL, USA); Stephen Gollomp (Wynnewood, PA, USA); David Greeley (Spokane, WA, USA); Stephen Grill (Elkridge, MD, USA); Philip Hanna (Edison, NJ, USA); Robert Hauser (Tampa, FL, USA); Neal Hermanowicz (Irvine, CA, USA); Zhigao Huang (Jacksonville, FL, USA); Bahman Jabbari (New Haven, CT, USA); Joseph Jankovic (Houston, TX, USA); Un Jung Kang (Chicago, IL, USA); Mark LeDoux (Memphis, TN, USA); Kenneth Levin (Ridgewood, NJ, USA); Peter LeWitt (Southfield, MI, USA); Anthony Nicholas (Birmingham, AL, USA); Robert Rodnitzky (Iowa City, IA, USA); Alok Sahay (Cincinnati, OH, USA); Harvey Schwartz (Hollywood, FL, USA); Burton Scott (Durham, NC, USA); Kapil Sethi (Augusta, GA, USA); Dee Silver (La Jolla, CA, USA); Carlos Singer (Miami, FL, USA); Lynn Struck (Des Moines, IA, USA); William Sunter (Melbourne, FL, USA); Daniel Truong (Fountain Valley, CA, USA); Alberto Vasquez (St. Petersburg, FL, USA); Maureen Wooten Watts (Dallas, TX, USA).
